# Revisiting the ICHD-3 criteria for headache attributed to mild traumatic injury to the head: Insights from the Toronto Concussion Study Analysis of Acute Headaches Following Concussion

**DOI:** 10.1177/03331024221099216

**Published:** 2022-05-11

**Authors:** Laura Kathleen Langer, Mark Theodore Bayley, David Wyndham Lawrence, Paul Comper, Alice Kam, Alan Tam, Cristina Saverino, Catherine Wiseman-Hakes, Lesley Ruttan, Tharshini Chandra, Evan Foster, Jonathan Gladstone

**Affiliations:** 1 KITE Research Institute at Toronto Rehabilitation Institute, University Health Network, Toronto, Canada; 2 Toronto Rehabilitation Institute, University Health Network, Toronto, Canada; 3 Department of Medicine, University of Toronto, Toronto, Canada; 4 Faculty of Kinesiology and Physical Education, University of Toronto, Toronto, Canada; 5 Department of Family and Community Medicine, Mt Sinai Hospital, Toronto, Canada; 6 Rehabilitation Sciences Institute and Institute of Health Policy, Management & Evaluation, University of Toronto, Toronto, Canada; 7 Toronto Western Hospital, University Health Network, Toronto, Canada; 8 Rehabilitation Science, Faculty of Health Sciences, McMaster University, Hamilton, Canada; 9 Graduate Department of Psychological Clinical Science, University of Toronto Scarborough, Toronto, Canada; 10 Department of Pediatrics (Division of Neurology), Hospital for Sick Children, Toronto, Canada; 11 Gladstone Headache Clinic, Toronto, Canada

**Keywords:** Post-traumatic headache, concussion, acute, headache, migraine, characteristics, epidemiology, diagnostic criteria, ICHD-3

## Abstract

**Background:**

There is limited prospective data on the prevalence, timing of onset, and characteristics of acute headache following concussion/mild traumatic brain injury.

**Methods:**

Adults diagnosed with concussion (arising from injuries not related to work or motor vehicle accidents) were recruited from emergency departments and seen within one week post injury wherein they completed questionnaires assessing demographic variables, pre-injury headache history, post-injury headache history, and the Sport Concussion Assessment Tool (SCAT-3) symptom checklist, the Sleep and Concussion Questionnaire (SCQ) and mood/anxiety on the Brief Symptom Inventory (BSI).

**Results:**

A total of 302 participants (59% female) were enrolled (mean age 33.6 years) and almost all (92%) endorsed post-traumatic headache (PTH) with 94% endorsing headache onset within 24 hours of injury. Headache location was not correlated with site of injury. Most participants (84%) experienced daily headache. Headache quality was pressure/squeezing in 69% and throbbing/pulsing type in 22%. Associated symptoms included: photophobia (74%), phonophobia (72%) and nausea (55%). SCAT-3 symptom scores, Brief Symptom Inventory and Sleep and Concussion Questionnaire scores were significantly higher in those endorsing acute PTH. No significant differences were found in week 1 acute PTH by sex, history of migraine, pre-injury headache frequency, anxiety, or depression, nor presence/absence of post-traumatic amnesia and self-reported loss of consciousness.

**Conclusions:**

This study highlights the very high incidence of acute PTH following concussion, the timing of onset and characteristics of acute PTH, the associated psychological and sleep disturbances and notes that the current ICHD-3 criteria for headaches attributed to mild traumatic injury to the head are reasonable, the interval between injury and headache onset should not be extended beyond seven days and could, potentially, be shorted to allow for greater diagnostic precision.

## Introduction

**Figure 1.**
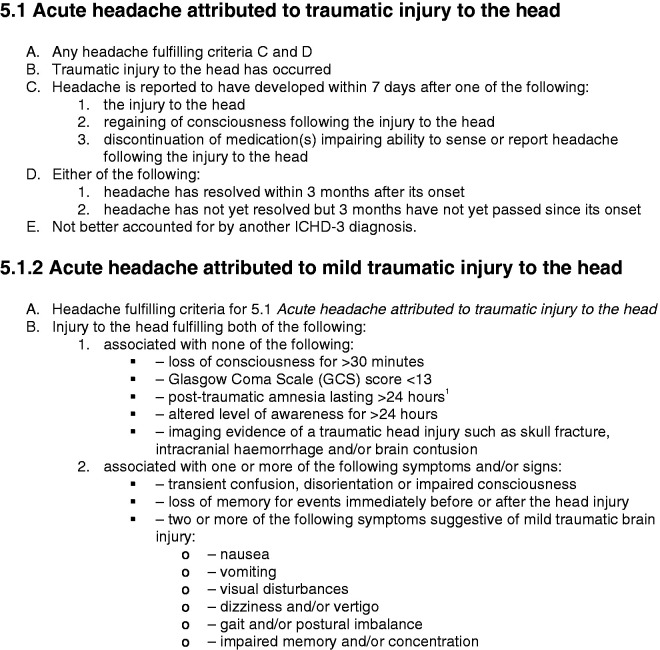
Diagnostic criteria for acute headache attributed to traumatic injury to the head and acute headache attributed to a mild traumatic injury to the head are outlined.

Concussions/mild traumatic brain injuries are very common neurological injuries that can cause physical, cognitive, and psychological dysfunction (1). The most common acute post-concussion symptom is headache (often referred to as post-traumatic headache or PTH). The acute headache following a concussion may persist for days, weeks or months post injury (2). The International Headache Society Classification of Headache Disorders Third Edition (ICHD-3) diagnostic criteria 5.1 for acute headache attributed to traumatic injury to the head and 5.1.2 acute headache attributed to a mild traumatic injury to the head are outlined in Figure 1 (3). When the headaches continues for more than 3 months post-injury, ICHD-3 defines them as persistent headaches attributed to traumatic injury to the head (3). To meet criteria for persistent headaches attributed to traumatic injury to the head, individuals would have first had to meet criteria for acute headaches attributed to traumatic injury to the head. These ICHD-3 criteria are largely based upon the consensus opinion of an expert panel rather than being evidence-based given the paucity of data in this area. Furthermore, these criteria do not identify any characteristic or defining headache quality, location, frequency, duration, associated symptoms, triggering or aggravating factors.

In terms of onset timing, the ICHD-3 criteria require that the headache develop within 7 days after injury (or after regaining consciousness or discontinuation of medication(s) impairing ability to sense or report headache). There has been debate regarding the validity of this time frame with some suggesting that the interval should be shorter and others suggesting that the interval should be extended out as far as thirty days post-injury or longer (3,6,7); indeed the appendix of the ICHD-3 proposes research criteria that extend the interval between traumatic injury to the head and headache onset out to ninety days (so called “delayed-onset” headache attributed to traumatic injury to the head). These divergent views demonstrate the need to evaluate and field-test the diagnostic criteria.

Despite its high prevalence, relatively little is known about acute and persistent PTH in terms of the epidemiology, headache characteristics, predictive factors for headache recovery or persistence, imaging correlates, and the role of genetics (4–8). The pathophysiology of acute PTH is not well understood. PTH results from physical forces that may precipitate headache de novo in a non-headache prone individual, initiate a new headache type in someone with pre-injury episodic or chronic headaches, or may exacerbate an existing primary headache disorder (2).

Unfortunately, there is an extremely limited number of relevant clinical trials upon which to make therapeutic decisions for PTH management. The hope is that with greater clarity in PTH diagnostic criteria, with renewed interest in PTH (9,10) and with promising new emerging therapies, appropriate patients can be enrolled in clinical trials and novel therapeutic agents and treatment strategies can be appropriately evaluated.

To date, there has been limited research on the clinical characteristics of acute headache following head trauma (5–7). Most prior research on PTH has been retrospective, looked at homogenous athlete, military or pediatric populations that do not optimally generalize to the general concussion population, or has focused on persistent PTH (11–15). Accordingly, this phase of the Toronto Concussion Study was designed to prospectively and comprehensively evaluate acute PTH within the first week post-concussion in a heterogeneous outpatient population. Specifically, the aim of this phase of the Study was to determine the prevalence of acute PTH in a general (non-motor vehicle accident and non-work-related injury) population, to ascertain the typical timing of onset and clinical characteristics of acute PTH and, in turn, determine the appropriateness of the ICHD-3 criteria for acute PTH. Another objective was to examine risk factors for the development of acute PTH. Subsequent analyses from the Toronto Concussion Study evaluate the risk factors for the persistence of PTH through the first three months post-concussion.

## Methods

### Participants

All participants were recruited from the Hull-Ellis Concussion and Research Clinic (“the Clinic”) at the Toronto Rehabilitation Institute of the University Health Network in Toronto, Canada between January 2016 and December 2018. All participants were referred from surrounding university-affiliated emergency departments (ED) with a diagnosis of concussion. Given the wide array of diagnostic criteria proposed for concussion (e.g., Berlin Consensus on Concussion in Sports (16), Ontario Neurotrauma Foundation Concussion Guidelines (17)), the diagnosis of concussion was left to the expert clinical judgment of the assessing ED physician. Participants were seen for an initial consultation at the Clinic, and after agreeing to participate in the study provided written informed consent within 7 days of their injury. 

Inclusion criteria included: aged between 17–85 years; physician diagnosis of concussion; Glasgow Coma Scale of 13–15; CT scan imaging negative or deemed unnecessary using a validated clinical decision rule; not admitted to hospital post-injury; deemed medically stable by clinic physicians; ability to be seen in clinic within 7 days of the injury and attend regular clinic visits; and able to comprehend English and complete questionnaires/measures in English. Participants were excluded if they had a third-party insurance claim-eligible mechanism of injury (i.e., workplace accident or motor vehicle collision); were symptomatic from a previous concussion that occurred <3 months ago; were unwilling or unable to comply with the trial follow-up requirements; or unable to provide informed consent.

### Research clinic assessments

Participants attending their first visit in the Hull-Ellis Clinic were invited to enroll in the Toronto Concussion Study. At baseline, participants underwent multiple assessments: a patient questionnaire that included demographic information, injury information, and prior health status; a Baseline Headache Questionnaire (assessing pre-injury headache history and initial post-injury headaches); a computerized version of the SCAT-3 symptom checklist (16); The Sleep and Concussion Questionnaire (SCQ) (18,19) (which is a Patient-Reported Outcome Measure (PROM) that describes and quantifies changes in sleep and wakefulness in response to brain injury), and the Brief Symptom Inventory (BSI) (20) psychological symptom questionnaire (administered at the week 2 visit). Participants also underwent neurocognitive testing and a physical examination by the treating physician. Patients were subsequently evaluated weekly for the first eight weeks after their injury followed by visits at week 12 and week 16. Data from follow-up visits were analyzed to assess for headache recovery trajectory and risk factors for persistence and will be presented in separate publications from the Toronto Concussion Study.

### Headache questionnaire

The Baseline Headache Questionnaire specifically inquired about (i) the frequency of pre-existing headaches of any type in the year prior to the accident (categorized as none, once every few months, once per month, twice per month, once per week, ≥3 times per week); (ii) pre-injury history of chronic headaches (headaches on more than 15 days per month) at any point pre-injury; (iii) pre-injury history of migraine at any time prior to the index accident; (iv) migraine frequency in the year before the index accident (classified as none, once every few months, once per month, twice per month, once per week, ≥2–3 times per week); and a family history of migraine.

Participants were asked whether they had developed a headache post-concussion. If yes, then participants were asked (i) when the headache began (i.e., immediately, later the day of the injury, the day after, 2–3 days after, 4–7 days after or more than 1 week after the injury. As this question was repeated at subsequent follow-up visits, more than 1 week after the injury was also an option to select); (ii) the frequency of the headaches; (iii) headache location and relationship to site of injury; (iv) headache characteristics; (v) headache frequency; (vi) headache quality; (vii) number of headache types and their characteristics; (viii) headache intensity: minimum, average and maximum intensity; (ix) associated symptoms; (x) aura symptoms; (xi) precipitating factors; (xii) aggravating factors; and (xiii) alleviating factors.

### Statistical analysis

All statistical analyses were performed on SAS 9.4 (SAS Institute, North Carolina, USA) for Windows. Alpha was set at 0.05. Continuous variables were analyzed using descriptive methods and group differences were determined using student’s t-test and ANOVA. Categorical variables were analyzed with Chi square contingency tables. All data are reported in accordance with STROBE requirements.

### Research ethics

This study was approved by the Research Ethics Board at University Health Network, study ID#15-9214. All participants provided written informed consent.

## Results

**Figure 2.**
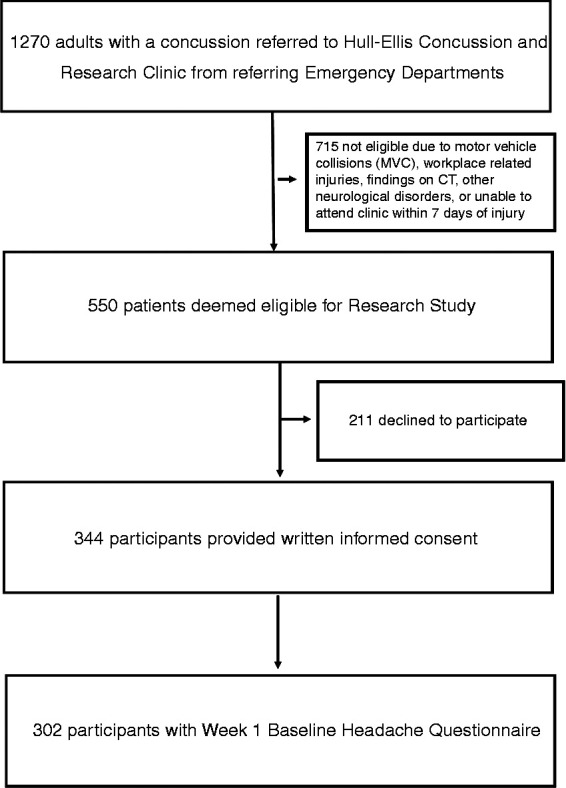
Flow diagram of the number of referrals, eligible potential participants, participants with written informed consent and Week 1 Baseline Headache Questionnaire.

Between January 2016 and December 2018, 1270 individuals were referred to the concussion clinic by various emergency departments. From this group, 555 individuals were deemed eligible to participate and 344 people provided consent to participate in this study. A total of 302 participants completed the Week 1 Headache Questionnaire and were included in this analysis (Figure 2).

Participants were seen at a mean of 5.3 (SD 1.8) days after their injury. The mean age was 33.6 (SD 13.0) years and 59% were female. At the first visit, the mean number of symptoms endorsed on SCAT-3 was 15.2 (SD 5.6) of 22 possible symptoms and mean symptom severity score was 45.2 (SD 28.8) of a possible 132 (reflecting a significant symptom severity burden). Participant demographics are presented in Table 1.

**Table 1.** Demographics of the total study population.

	N (%)
Total Study population	302
Age (years)	33.6 (SD 13.0)
Sex (Female)	178 (59%)
Loss of consciousness (LOC)	68 (23%)
Post-traumatic injury (PTA)	48 (16%)
Mechanism of injury	
Falls	98 (32%)
Sports related	80 (26%)
Striking blow	34 (11%)
Assault	27 (9%)
Pedestrian	26 (9%)
Bicycle related	37 (12%)
Most frequent highest education achieved	Undergraduate degree (16 years education)
History of anxiety	68 (23%)
History of depression	45 (15%)
History of chronic headache	38 (13%)
History of migraine	116 (38%)
Family history of migraine	108 (36%)
Pre-injury prescription medication for migraine/headache	12 (4%)
Pre-concussion Frequency of Headache	
>2–3 times a week	25 (8%)
Weekly	26 (9%)
Twice a month	47 (16%)
Monthly	49 (16%)
Every few months	113 (37%)
Less frequent/never	42 (14%)

### Acute post-traumatic headache characteristics

*a) Headache prevalence*

At the Week 1 clinic visit, 277 individuals (92%) endorsed that they experienced a post-injury headache(s). Headache characteristics are presented in Table 2.

**Table 2.** Headache characteristics.

Headache endorsement	277 (92%)
Frequency
Continuous/constant	69 (25%)
Daily	163 (59%)
4–7 days/week	12 (4%)
2–3 days/ week	6 (2%)
1 day/ week	5 (2%)
Once every 2 weeks	0
Once a month	0
Duration	
Continuous/constant	97 (35%)
Multiple days	12 (4%)
1 Day	22 (8%)
4–8 Hours	26 (9%)
2–3 Hours	43 (16%)
1 Hour	31 (11%)
30 Minutes	40 (14%)
1 Minute	6 (2%)
Location of pain	
Temple	138 (50%)
Forehead	135 (49%)
Behind the eye(s)	134 (49%)
Back of head	128 (46%)
Neck	94 (34%)
Top of head	84 (30%)
Whole head	53 (19%)
Same site as injury	112 (44%)
Headache quality	
Pressure/tension	190 (69%)
Throbbing	61 (22%)
Stabbing	9 (3%)
Other	16 (6%)
Headache with visual aura	33 (12%)
Headache intensity	
Maximum headache pain rating (10 point scale)	6.2 (SD 2.3)
Mean headache pain rating (10 point scale)	4.6 (SD 2.0)

*b) Timing of headache onset*

From the cohort of 277 patients who experienced headache, 260 (94%) indicated that their headache onset was the day of the concussion or by the following day post-injury. Of those 262 individuals who indicated that their headaches began within one day of the injury, 147 (57%) indicated that their headache began “immediately after injury”, 69 (26%) selected “later the same day as the injury”, and 46 (17%) endorsed headache onset the day after injury; 16 (6%) individuals noted that they had headache onset more than 48 hours after injury but within 7 days of injury.

Of the 25 individuals who did not endorse headache at Week 1, only four (1% of the study population) endorsed a new headache at the Week 2 visit; however, all four of these participants indicated that their headache began within 2–3 days post-injury. Upon evaluation of the database, these four patients were all initially evaluated early in the first week post-injury (days 2–4 post injury) and, as such, perhaps they were seen prior to headache onset or their Week 2 data reflects a bit of recall bias for the early timing of onset of the acute PTH. Further, of the remaining 21 participants who did not endorse PTH at Week 1 or Week 2, none endorsed a new headache during the 12-week follow-up period. Notably, of these 21 participants, there were five patients who were lost to follow-up before Week 8 and 13 patients were discharged before Week 8 as they were deemed by study investigators to be fully recovered with complete symptom resolution.

*c) Headache frequency*

At the initial clinic, most participants with acute PTH endorsed a daily headache (n = 232, 84%) which was either intermittent (n = 163, 59%) or continuous (n = 69, 25%).

*d) Headache duration*

The most commonly selected durations were: continuous (n = 97, 35%), lasting 2–3 hours (n = 43, 16%), or lasting 30 minutes (n = 40, 14%).

*e) Headache location*

Amongst the multiple head pain locations that could be selected simultaneously on the questionnaire (including specific locations, unilateral or holocranial), the most common location of head pain selected was the temple (n = 138, 50%), followed by the forehead (n = 135, 49%), behind the eyes (n = 134, 49%), and back of head (n = 128, 46%). Only 94 (34%) endorsed neck pain. Holocranial pain was endorsed by 53 participants (19%). Notably, there was no association between the location of pain and the site of injury (p = 0.9).

*f) Headache intensity*

The mean maximal pain intensity was 6.2/10 (SD 2.3). Mean average pain intensity was 4.6/10 (SD2.0).

*g) Headache characteristics*

At Week 1, 190 (69%) selected “pressure/squeezing/tightness” as their primary headache phenotype while 61 (22%) indicated “throbbing/pulsing” as their primary phenotype; 9 (3%) endorsed “stabbing-needle-like” pain and 16 (6%) indicated “none of the above”. Having more than one headache phenotype was endorsed by 41%.

*h) Associated symptoms*

The most frequent symptoms concomitant with headache(s) included: difficulty concentrating/thinking (n = 208, 75%), sensitivity to lights (n = 205, 74%), sensitivity to sound (n = 199, 72%), fatigue (n = 199, 72%), light-headedness (n = 191, 69%), and nausea (n = 152, 55%). Trigeminal autonomic symptoms were less commonly reported (conjunctival injection (n = 50, 18%), lacrimation (n = 25, 9%), ptosis (n = 14, 5%), rhinorrhea/congestion (n = 44, 16%). Whether or not the trigeminal autonomic symptoms were unilateral or bilateral and whether or not they were ipsilateral to a unilateral headache was not evaluated. 33 individuals (12%) reported visual aura-type symptoms preceding or accompanying their headaches.

*h) Headache triggers and aggravating factors*

For those without continuous headache, the following were identified as the most common factors triggering/precipitating headaches: thinking/concentration (n = 103, 34%), loud sounds (n = 93, 31%), busy/crowded environments (n = 91, 30%), bright lights (n = 88, 29%), and screens (n = 86, 28%).

The following were identified as the most common aggravating factors: thinking/concentration (n = 180, 60%), bright light (n = 179, 59%), computer screens (n = 175, 58%), loud sounds (n = 173, 57%), and crowded/busy environments (n = 164, 54%).

**Figure 3.**
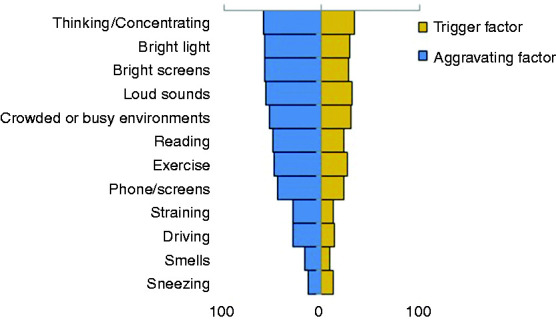
Percentage of people endorsing headache triggering factors (blue) and headache aggravating factors (yellow).

Thinking/concentrating was reported as both the most common trigger and the most common aggravating factor at Week 1. Figure 3 outlines the most common triggering and aggravating factors.

*i) Concomitant post-concussion symptoms*

Participants endorsing ongoing headache(s) at their initial concussion clinic visit had significantly higher SCAT-3 symptom severity scores (mean, 46.9; SD, 28.8; 95%CI 43.5 – 50.3) than participants not endorsing headaches (mean, 26.6; SD, 21.3; 95%CI 17.8–35.4; p = 0.0007) (Table 3). Furthermore, participants endorsing a headache at the initial visit endorsed a greater number of symptoms on the SCAT-3 (number of symptoms, 15.6; SD, 5.4; 95% CI 14.9 – 16.2) than those not endorsing a headache (number of symptoms, 11.1; SD, 6.2; 95% CI 8.6 – 13.7; p = 0.0001).

**Table 3.** Differences in demographic, injury factors, Sport Concussion Assessment Tool (SCAT) symptom severity inventory, Brief Symptom Inventory (BSI) scores, and sleep change following concussion based upon acute post-traumatic headache at Week 1.

	Overall study population (302)	Endorsing headache at Week 1	No headache at Week 1
Endorsing headache at first visit		277 (92%)	25 (8%)
Age (years)	33.6 (SD 13.0)	33.0 (SD 12.8)*	39.2 (SD 13.8)
Sex (female)	178 (59%)	166 (93%)	12 (48%)
SCAT symptom severity score	45.2 (SD 28.8)	46.9 (SD 28.8)**	26.6 (SD 21.3)
SCAT number of symptoms	15.2 (SD 5.6)	15.6 (SD 5.4)**	11.1 (SD 6.2)
Loss of consciousness (LOC)	68 (23%)	61 (89%)	7 (28%)
Post traumatic amnesia (PTA)	48 (16%)	46 (96%)	2 (8%)
History of chronic headache	38 (13%)	38 (100%)	0
History of migraine	116 (38%)	108 (93%)	8 (32%)
Family history of migraine	108 (36%)	99 (92%)	9 (36%)
History of anxiety	68 (23%)	66 (97%)	2 (8%)
History of depression	45 (15%)	42(93%)	3 (12%)
BSI global score	55.7 (SD 10.1)	56.4 (SD 10)*	48.5 (SD 7.5)
BSI somatic subscale	56.9 (SD 9.3)	57.5 (SD 9.3)*	51.4 (SD 7.4)
BSI anxiety subscale	54.3 (SD 11.1)	55.1 (SD 11.2)**	46.4 (SD 7.0)
BSI depression subscale	52.7 (SD 10.4)	53.4 (SD 10.4)*	46.6 (SD 7.6)
Sleep questionnaire score	12.6 (SD 6.1)	13.0 (SD 6.1)**	8.1 (SD 4.8)
Change in sleep			
None	58 (19%)	49 (18%)	9 (36%)
Mild change	105 (35%)	96 (35%)	9 (36%)
Moderate change	89 (30%)	84 (30%)	5 (20%)
Severe change	49 (16%)	47 (17%)	2 (8%)
Type of change (n=240)			
Sleeping less	53 (22%)	50 (22%)	3 (12%)
Sleeping more	144 (60%)	136 (61%)	8 (32%)
Lest restful sleep	43 (18%)	38 (17%)	5 (20%)

*p < 0.05.

**p < 0.01.

### Headache associated factors

*a) Age and sex*

There was no significant difference in Week 1 headache endorsement by sex (p = 0.25). The age of the participants endorsing acute PTH (mean age, 33.0 years; SD, 12.8; 95%CI 31.5–34.5) was significantly lower compared to participants who did not endorse PTH (mean age, 39.2 years; SD, 13.8; 95%CI 33.5–44.9; p = 0.02).

*b) Pre-injury headache history*

Pre-injury frequency of headache did not predict endorsing a new headache at Week 1 (p = 0.91). Those with a prior history of chronic daily headaches at any point in their life prior to the index concussion were not more likely to endorse headaches in the first week post-concussion (n = 38, 13%; chi square value = 2.90, p = 0.09)

There was no significant difference in the prevalence of acute PTH endorsement at the first clinic visit post-injury in individuals with a history of migraine (n = 116, 38%) compared to those without a history of migraine (chi square value = 0.4,7 p = 0.49).

*c) Pre-injury mental health*

There was no significant difference in acute PTH endorsement at the first clinic visit post-injury based upon premorbid anxiety (chi square value = 3.29, p = 0.07) or history of depression (chi square value = 2.74, p = 0.10).

*d) Presence/absence of loss of consciousness or post traumatic amnesia*

There was no significant difference in acute PTH endorsement at the first clinic visit post-injury based upon loss of consciousness at the time of injury (as determined by treating physician) (chi square value = 0.23, p = 0.63), or presence of self-reported post-traumatic amnesia (chi square value = 1.16, p = 0.26).

*e) Sleep factors*

Changes in sleep were noted in 81% (n = 240) of those with acute PTH at the first visit post-injury whereas only 62% (n = 15) of those not endorsing PTH also experienced a change in their sleep after injury.

Patients with acute PTH at their first clinic visit, had significantly higher scores on the Sleep and Concussion Questionnaire (mean 13.0; SD, 6.1; 95% CI 12.3–13.7) than those without PTH (mean, 8.1; SD, 4.8; 95% CI 6.1–10.1; p = 0.001). Sleeping more than pre-injury was most commonly reported by 61% (n = 136) in the acute PTH group compared to 43% (n = 8) in the group without PTH (p = 0.001). There was an increased need for daytime naps in those with acute PTH (n = 186, 83% cf. n = 10, 60% non-PTH group, p = 0.0001). A total of 211 (94%) of those with acute PTH self-reported daytime sleepiness compared to 13 (80%) of those without acute PTH (p = 0.001) and 141 (63%) with acute PTH had difficulty staying awake whereas 7 (46%) without headache had trouble staying awake during the day (p = 0.001).

*f) Psychological factors*

Participants endorsing acute PTH at their initial visit had significantly higher psychological symptoms on the BSI Global Score with mean 56.4 (SD, 10; 95%CI 55.1–57.8) vs a mean of 48.5 (SD, 7.5; 95%CI 44.9–52 (p = 0.0006)) in those without acute PTH. The higher BSI Global Score in those with acute PTH at Week 1 included higher subscale scores in (i) Anxiety (p < 0.0001), mean 55.1 (11.2 SD 95%CI 53.6–56.6) vs a mean of 46.4 (7 SD, 95% CI 43.1–49.6]), (ii) Somatic (p = 0.0019), mean 57.5 (9.3 SD, 95%CI 56.3–58.8) vs mean of 51.4 (7.4 SD 95% CI 47.9–54.8), and (iii) Depression (p = 0.005), mean 53.4 (SD10.4 95%CI 52–54.8) vs mean of 46.6 (7.6 SD 95%CI 43–50.2).

## Discussion

The current ICHD-3 guidelines for acute headache attributed to a mild traumatic injury to the head are based upon expert opinion rather than evidence. The Toronto Concussion Study is one of the few studies to systematically and prospectively evaluate acute headaches attributed to head injury within the first week post-concussion in a general (non-compensable injury) population.

Out of 302 patients with diagnosed concussions by ED physicians, almost all patients (92%) examined within a week post-injury at the Hull-Ellis Concussion and Research Clinic post-concussion endorsed acute PTH. Those presenting with acute PTH at their initial clinic visit all met ICHD-3 criteria for acute headaches attributed to mild traumatic injury to the head and were carefully and thoughtfully evaluated.

The results of our study validate the current ICHD-3 criteria for acute headaches attributed to head injury. The Toronto Concussion Study provides meaningful evidence to support the ICHD-3 assertion there are no defining clinical characteristics of acute PTH (i.e., pain frequency, duration, location, quality, associated symptoms, provoking and aggravating factors are heterogeneous). The most reported headache frequency was daily and intermittent (59%) or daily and continuous (25%). Headache location was highly variable and was not correlated with the site of injury. In terms of primary headache quality, the most common quality was pressure/squeezing/tightening in 69% of patients whereas throbbing/pulsing was endorsed by 22%. Very few patients had neuropathic-type head pain (stabbing). 41% of patient had more than one headache phenotype. Commonly associated symptoms included photophobia (74%), phonophobia (72%) and nausea (55%). Trigeminal autonomic symptoms occurred in some patients but were much less common.

Given the persistence of continuous headaches in many patients and the clear overlap between symptoms of concussion (i.e., nausea, photo/phonophobia) and symptoms of headache in the acute phase post-injury, it is challenging to accurately characterize individual patients’ headache phenotypes as tension-type or migraine and, as such, we chose to describe the acute PTH characteristics noted rather than try to assign primary headache phenotype labels to individual patients.

Our study identified that almost all individuals (94%) with acute PTH experience headache onset the day of the concussion or the following day and only a small minority develop headaches later within the first seven days post-injury. Further, no patients in our study endorsed a new onset acute PTH occurring beyond one-week post-injury. Accordingly, this suggests that the current ICHD-3 interval for headache onset should not be extended beyond seven days. Furthermore, the results raise the spectre of reducing the interval between injury and headache onset to less than seven days to improve diagnostic precision.

Certainly, we have all seen patients who endorse headaches that begin beyond seven days post-injury; however, it is our belief that such headaches should not be considered to be a *direct* sequelae of the head injury (i.e., meet criteria for acute or persistent headaches attributed to traumatic injury to the head). It is important to recognize that there are innumerable precipitants for headache and many of these provocative factors are experienced by individuals following concussion/traumatic brain injury (e.g., adverse psychological reactions to the injury-related circumstances, depressed or anxious mood, acute psychosocial or financial stressors, workplace-related stressors, narcotic medication use for concomitant polytrauma, sleep deprivation, interrupted sleep, altered sleep-wake cycles, etc.). It is highly problematic for the ICHD-3 criteria for headaches attributed to traumatic injury to the head to impugn a head injury to be directly causally related to headache onset when the headache begins more than one-week post-injury as inclusion of such individuals in ICHD diagnostic criteria is not substantiated by prospective studies. Further, inclusion of individuals with delayed onset headache is likely to effectively “muddy the waters” leading to heterogeneous patient populations making epidemiologic, clinical and pharmacologic research uninterpretable.

In the Toronto Concussion Study, there were significant differences between those with acute headache post-concussion and those without acute headache. Specifically, when compared with those concussion patients who did not experience a new headache in the first week post-concussion, those with acute PTH had significantly higher: (i) number of symptoms and symptom severity scores on the SCAT-3, (ii) psychological disturbances on the Brief Symptom Inventory and (iii) sleep disturbances on the Sleep and Concussion Questionnaire, consistent with previous research on PTH.^21–23^

In our study, no significant differences in acute PTH endorsement within the first-week post-injury were found based on injury parameters (self-reported loss of consciousness or post-traumatic amnesia), sex and pre-accident factors including headache frequency, migraine, anxiety or depression.

Upcoming analyses from the Toronto Concussion study will explore the trajectory of recovery from acute PTH, risk factors for development of persistent PTH and characteristics of persistent PTH.

Our study is one of the few to prospectively evaluate acute headache following concussion/mild traumatic brain injury. Lucas and colleagues (6) prospectively enrolled 212 patients with head injuries and assessed PTH prevalence and provided characterization of the headaches. Their study population differed from the Toronto Concussion Study in that patients were recruited from a Level 1 trauma center in Seattle, Washington, the patients were overwhelming male (76%), 58% were injured in vehicle-related crashes (i.e., potentially compensable injuries) and data was collected “usually while hospitalized for observation or other body system injuries.” While headache prevalence was documented at week 1, headache characteristics were not documented until the three-month post-injury follow-up. In contrast, our study population sought to exclude individuals with potentially compensable injuries to try to avoid any litigation-bias or other factors that may be unique to the compensable head injury population.

In the study by Lucas and colleagues, a lower percentage of patients (54%) endorsed a new or worse headache after the injury at 1-week post-injury versus the Toronto Concussion Study (92%). In the study by Lucas and colleagues, 59% of those who did not endorse a new or worse headache at week 1 endorsed a new or worse headache (compared to pre-injury) at the three-month follow-up (the first follow-up after the initial visit); Lucas and colleagues utilize these results to suggest that the time interval accepted by the ICHD-3 for headaches attributed to traumatic injury to the head should be increased beyond seven days to a longer time period. This contrasts with our study and the study of Lieba-Samal and colleagues (7) wherein delayed onset PTH was not identified.

Lieba-Samal and colleagues recruited 100 patients with acute mild head injury presenting to the department of trauma surgery at the Medical University of Vienna. The patients were primarily male (66%), and 38% were admitted to hospital post-injury. Patients underwent a detailed telephone interview between days 7 and 10 post injury and again between 90–100 days post-injury. The prevalence of acute PTH was 66%. For those with acute PTH, 92% developed their headache within 48 hours after the trauma. The presence of acute PTH was related to chronic pain (excluding headache), pre-existing episodic headache, number of posttraumatic symptoms, anxiety and depression. None of the patients continued to have persistent PTH when assessed at 90–100 days and none of the patients who did not endorse headache at 7–10 days post-injury endorsed the emergence of a new headache at 90–100 days post-injury.

In contrast to our study, Lieba-Samal and colleagues identified a lower number of patients who presented with acute PTH (66% vs 92%). Similar to our study, a very high percentage of those with acute PTH developed their headache within the first 48 hours of the head injury (92% vs 94%). Similar to our study, headache frequency and location was heterogeneous. Headache qualities reported were similar between Lieba-Samal’s study and the Toronto Concussion Study: pressure (69% vs 69%) and pulsating (34% vs 22%) as was mean headache intensity (4.2/10 vs 4.6/10). Similarly, associated symptoms were similar between Lieba-Samal’s study and the Toronto Concussion Study: nausea (42% vs 55%) and photo/phonophobia (55% vs 72–74%). Also similar to the Toronto Concussion Study, Lieba-Samal and colleague identified that those who developed acute PTH following head injury were more likely to have a higher number of post-concussive symptoms, and a greater degree of anxiety and depressive symptoms.

Unlike our study, Lieba-Samal and colleagues were able to identify a number of predictors for the development of acute PTH. It is likely that we were not able to identify any statistically significant predictors for the development of acute PTH because almost all participants (92%) developed acute PTH limiting statistical power.

### Limitations

Limitations of this study include the fact that the inclusion criteria did not specify formal diagnostic criteria for concussion and relied on individual physician diagnosis of concussion. However, it is noteworthy that the ICHD-3 also does not include formal diagnostic criteria for concussion and, indeed, the ICHD-3 criteria only delineate between mild and moderate to severe traumatic injuries to the head. Another limitation of this data is that evaluation of headaches was performed on a weekly basis rather than via daily electronic record; given difficulty tolerating screen use post-concussion, daily symptom recording was not felt to be prudent and it was felt that the initial assessment within 7 days post-injury and the regular weekly follow-up assessments thereafter were sufficient to accurately capture headache characteristics. Baseline pre-injury information was provided by self-report rather than formal chart review potentially negatively impacting evaluation of predictors for the development of acute PTH. Evaluation of acute PTH characteristics was completed by use of questionnaire rather than physician evaluation; it is possible that more precise physician evaluation would have further refined evaluation of headache characteristics and allowed for more precise phenotyping of individuals with tension-type, migraine or cervicogenic; however, as noted above, given the significant potential overlap between symptoms due to concussion and, potentially, whiplash injury, it was not felt that attempted precise characterization of acute PTH phenotypes was appropriate. A small number of patients in the study were lost to follow-up or were discharged from clinic (due to symptom resolution) between 3–12 weeks post injury and it is possible that some of those patients may have gone on to develop the onset of a new headache in a delayed manner; however, the number of such patients was a small percentage of the overall study population and the relationship between concussion and any delayed onset of headache remote from a concussion in an otherwise fully recovered individual would be tenuous. Finally, the current study excluded individuals who sustained injuries that were potentially compensable and, as such, results may not be fully generalizable to those sustaining work-related or motor vehicle related accidents.

## Conclusions

Consistent with the prior study of Lieba-Samal (7), the overwhelming majority of concussed individuals followed prospectively develop acute PTH on the day of the injury or by the following day. Delayed-onset PTH was not observed. PTH characteristics are highly variable with no prototypical location, quality, duration, associated symptoms or aggravating/provoking factors. Those who experienced acute PTH had a higher number and overall burden of symptoms and a greater likelihood of concomitant sleep and psychological disturbances. Given the high prevalence of acute PTH in our study, we were unable to identify any predictive factors for the development of acute PTH.

The findings of the Toronto Concussion Study together with the findings of Lieba-Samal and colleagues suggest that current ICHD-3 criteria for acute headache attributed to traumatic injury to the head are reasonable and appropriate and the interval between injury and headache onset should not be extended beyond seven days. Indeed, given that 94% of the patients in this study had acute PTH onset the day of their concussion or by the next day, consideration could be given by the ICHD to reducing the accepted interval for headache onset post-injury from seven days to a shorter interval to improve diagnostic precision.

## Clinical implications

- Headache is an extremely common symptom after concussion and onset is typically within the first 24 hours post-injury
- The characteristics of acute headache attributed to traumatic injury to the head are highly variable with no prototypical frequency, duration, location, quality, associated symptoms, triggering or aggravating factors.
- There were no identified predictive factors for development of acute PTH (as almost all participants experienced headaches within the first week).
- Those who experienced acute PTH had higher overall burden of symptoms and symptom severity, and greater likelihood of sleep and psychological disturbances.
- ICHD-3 5.1 criteria for acute headache attributed to traumatic injury to the head are reasonable and appropriate; the accepted interval between the time of the injury and headache onset should not be extended beyond 7 days and consideration could be given to reducing the interval to 48 hours to improve diagnostic precision.
